# Effect of tetragonal to cubic phase transition on the upconversion luminescence properties of A/B site erbium-doped perovskite BaTiO_3_†

**DOI:** 10.1039/c8ra09783f

**Published:** 2019-01-18

**Authors:** Hyeongyu Bae, Kang Taek Lee

**Affiliations:** Department of Chemistry, Gwangju Institute of Science and Technology Cheomdangwagi-ro 123, Buk-gu Gwangju Republic of Korea ktlee@gist.ac.kr

## Abstract

With the increasing number of applications for upconversion materials, a more detailed understanding of the intrinsic mechanisms of their optical processes is required. Thus far, various lanthanide-doped host materials or nanoparticle systems have been investigated as representative upconversion systems owing to their major advantage as optical probes. As for the energetics of upconversion and the associated upconversion pathways, the role of the host material is very important because it provides a unique microscopic environment; for example, a unique local lattice structure in the case of crystalline samples. In general, the upconversion luminescence intensity decreases as a function of temperature owing to thermally accelerated multiphonon relaxation. Here, we report that the temperature dependence of the upconversion luminescence efficiency is affected differently in an Er^3+^-doped perovskite material, barium titanate (BaTiO_3_, BT), than in a general system. We show that Er^3+^ doped at the A (Ba^2+^) and B (Ti^4+^) sites of tetragonal phase BT, referred to as A-BT and B-BT, respectively, show different upconversion behaviors. The slope of the plot of the upconversion emission intensity as a function of temperature changed significantly in case of B-BT, but not for A-BT. This anomalous behavior of Er^3+^-doped BT is attributed to the phase transition (at ∼120 °C) of BT from tetragonal to cubic phase. Essentially, the temperature-dependent upconversion luminescence trend depends on the doping sites of Er^3+^, *i.e.*, at A or B sites in BT, which is explained by the difference in the symmetry of the crystalline structure with different crystal phase surrounding the Er^3+^ ions.

## Abstract 2

At the critical temperature, B-site doping with erbium shows abnormal upconversion efficiency as a function of temperature in perovskite crystals, which was attributed to the symmetry of the crystalline structure around the erbium ions.

## Graphical abstract

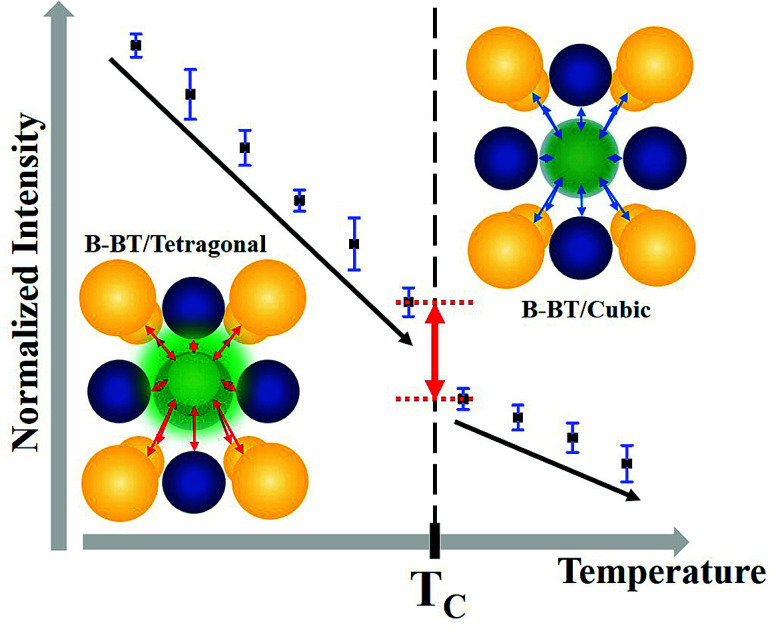

## Introduction

Upconversion (UC) is a useful photophysical phenomenon and has been applied in various fields, such as the solar cell industry,^1–3^ biological imaging^4,5^ or pH sensing,^6–8^ and temperature sensing.^9–11^ The range of spectral emissions of upconversion luminescence (UCL) from upconversion materials (*e.g.* upconverting nanoparticles, UCNPs) is wide and extends throughout the UV, visible, and IR regions, depending on the type of lanthanide ion doped in the host material. UCNPs increase the energy of the incident photons through the upconversion process. Although various mechanisms have been suggested, every mechanism is based on multiphoton absorption aided by the lanthanide electronic states with long lifetimes ranging from microsecond to millisecond timescale. Moreover, the ladder-like evenly spaced electronic states enable efficient absorption of photons of a single wavelength. The relevant transitions generally involve forbidden 4f–4f transitions, which are extremely stable because of the shielding effect by 5s or 5p electrons. The shielding effect also produces sharp 4f–4f transition bands due to weak coupling of the lattice and weak crystal field.^12^ Despite these advantageous features, the relatively low UC quantum yield of UC materials limits their application in many fields, especially in biomedical pharmacology, where a small dose of photons is preferred. Therefore, numerous research efforts are underway to enhance the efficiency of UCL by various methods such as doping of different sized ions^13,14^ or making a core–shell structure.^15,16^

The intensity of the 4f–4f transition was first described by Judd^17^ and Ofelt using the electric dipole mechanism.^18^ Because the intra-4f transitions are parity forbidden, which results in weak transition dipole moment, the lanthanide ions show low upconversion efficiency and weak absorption or emission.^19^ Judd–Ofelt's theory is based on the assumption that mixing of the ground 4f^*N*^ state with higher electronic configurations has odd terms in the crystal field Hamiltonian.^17,18,20^ The luminescence intensities of lanthanide ions are significantly influenced by their site symmetry.^21^ In general, a local crystal structure with low symmetry around the activator (emitting ion) leads to enhanced mixing between the 4f state and higher electronic configuration; consequently, the upconversion efficiency is enhanced.^12^ Various studies have been published concerning the problem of low upconversion efficiency. Wisser *et al.* showed that site-specific doping of gadolinium (Gd) and lutetium (Lu) at Y sites in NaYF_4_ UCNPs leads to a local symmetry-distorted lattice around the activator (Er^3+^).^14^ Furthermore, the radiative efficiency of transitions from the excited states to the ground state (^4^I_15/2_) have been enhanced substantially. With an increasing odd-parity interaction of the Er^3+^ ion with the host crystal, the f orbitals and higher electronic configurations are mixed significantly, leading to enhanced emission intensity.^12^ Thus, in the absence of such non-symmetrical crystal fields, the degenerate f orbitals of lanthanide ion would not produce any radiative transition.

**Fig. 1:**
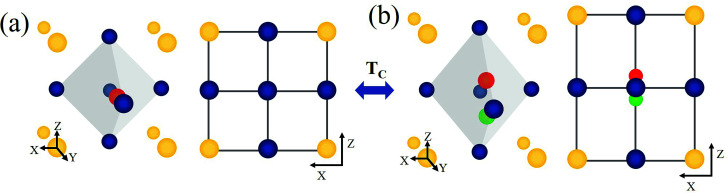
Schematic of a BaTiO_3_ unit cell (A site Ba^2+^ ion: yellow, B site Ti^4+^ ion: red or green, O^2−^ ion: blue): (a) BaTiO_3_ unit cell in the cubic phase, 3D view (left) and (001) plane view (right). (b) BaTiO_3_ in the tetragonal phase with different orientations of polarization (green and red Ti^4+^), 3D view (left) and (001) plane view (right). In case of tetragonal phase, the centered Ti^4+^ ion moves along the [001]-direction. *T*_c_ represents the Curie temperature, the temperature at which reversible phase transition occurs between the two phases.

BaTiO_3_ (BT) and related advanced ABO_3_ perovskite materials have been widely investigated as host materials for upconversion.^22–24^ BT is a versatile perovskite material characterized by a high dielectric constant, high charge storage capacity, electrical insulating property, and optical transparency in the visible range.^25–28^ The cubic and tetragonal crystal structures of BT are shown in Fig. 1. In the cubic phase, Ba^2+^ is located at the corner of the cube (A site), O^2−^ is located at the center of the face (X site), and Ti^4+^ is located at the center of the body (B site).^29^ In the tetragonal phase structure, the unit cell is stretched along one axis (*z*-axis), and the position of the Ti^4+^ ion at the center of the body is slightly shifted upward or downward in the same axis (*z*-axis).^30–32^ This phenomenon leads to the ferroelectricity of BaTiO_3_, and the unit cell shows polarization. The Curie temperature of BT is ∼120 °C,^31,33^ above which BT undergoes a transition from the tetragonal phase to the cubic phase. In addition, as shown in the infrared spectrum of BT (Fig. S1†), BT is confirmed to be an appropriate host with a low maximum phonon energy. In this study, we prepared two site-specific Er^3+^-doped BT samples, that is, Er^3+^ doped at A sites of BT (A-BT), and Er^3+^ doped at B sites of BT (B-BT), separately *via* solid-state reactions. In addition, we investigated the temperature dependent upconversion luminescence properties of the powders (A-BT and B-BT). We found that the UCL changed similarly as a function of temperature, but A-BT and B-BT show different behaviors at the Curie temperature. This is the first demonstration of the relationship between UCL and the phase transition of the BT host.

Purely tetragonal Er^3+^-doped BT powders were synthesized *via* solid-state reactions. Since the solid-state reaction is an ion-size-dependent doping method that uses accurately calculated stoichiometric quantities of reagents, the solid-state reaction is suitable for doping the ion at a specific site in the crystal.^34^ In this study, Ba_0.98_TiO_3_:Er_0.02_ (A-BT) and BaTi_0.98_O_3_:Er_0.02_ (B-BT) were synthesized, because the fraction of Er^3+^, 2%, turned out to yield the highest emission intensity, clearly at 525 and 550 nm as shown in Fig. S2.† This results is the same result as the previous study.^35^ We anticipate that if 1% and 3% Er^3+^ ions are site specifically doped at A site and B site of BT, the phase transition dependent upconversion luminescence intensities decay tendency will be similar with 2% Er^3+^ ion doped BT (A-BT, B-BT). To increase the surface area of the reaction, sodium chloride (99.5%, TCI) with a melting point of 801 °C was used as the flux. Barium carbonate (≥99%, Sigma-Aldrich) and titanium(iv) oxide (99.8%, Sigma-Aldrich) were used as barium and titanium source, respectively. Erbium oxide (99.9%, Sigma-Aldrich) was used as the Er^3+^ source. The molar ratios of the reagents vary depending on the concentration of the doped ions (see Table 1). The amount of sodium chloride was adjusted at NaCl/BaTiO_3_ = 1. A stoichiometric amount of the precursor powder material was ground for 1 h with an agate mortar and pestle. After that, the mixed reagents were loaded in a furnace and sintered for 5 h at 1100 °C (heating rate: 5 °C min^−1^) The product, tetragonal BT, is a white delicate powder that turns slightly pinkish after Er^3+^ doping. The structures of the as-prepared A-BT and B-BT powders were characterized by X-ray diffraction (XRD, X'Pert PRO Multi-Purpose X-ray Diffractometer, PANalytical) using Cu Kα radiation in the 2*θ* range of 20° to 80°. Further, X-ray diffraction (D/MAX-2500, RIGAKU) between 65 and 155 °C (at intervals of 10 °C) under vacuum conditions was also performed using Cu Kα radiation in the same 2*θ* range.

**Table 1:** Stoichiometric amounts needed for solid-state reaction

Material	Dopant ions	Dopant concentration	Concentrations of species (mol%)			
			Ba	Ti	Er	NaCl
t-BaTiO_3_	Er^3+^	2% (on Ba^2+^ site)	0.98	1	0.02	1
t-BaTiO_3_	Er^3+^	2% (on Ti^4+^ site)	1	0.98	0.02	1

**Fig. 2:**
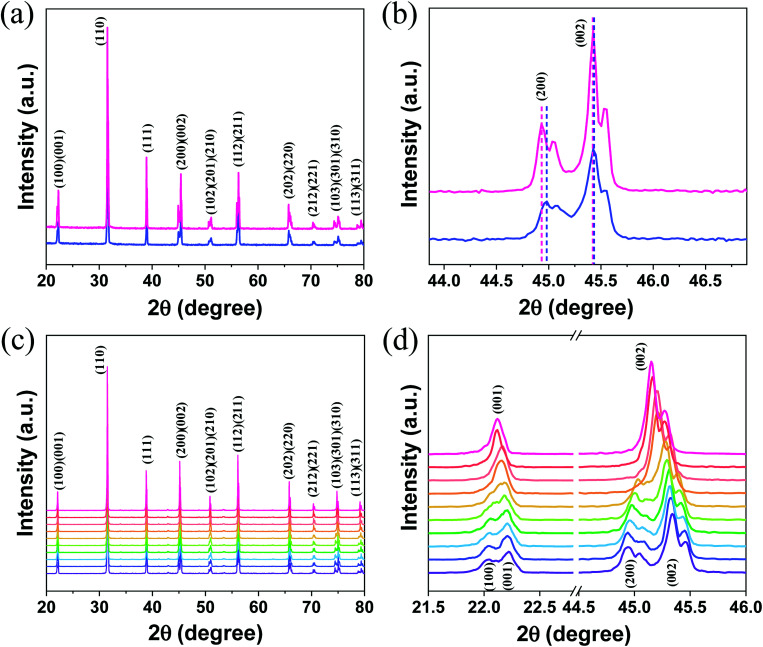
(a) Room-temperature XRD patterns of tetragonal A-BT (blue) and B-BT (pink) and (b) magnification of the 44.0–46.5° region. (c) High-temperature XRD patterns of B-BT and (d) magnification of the 21.5–23° and 44.5–46° regions. Purple to pink: 65–155 °C with an interval of 10 °C.

The room-temperature XRD patterns of A-BT and B-BT are shown in Fig. 2a. The blue and pink diffraction patterns are from A-BT and B-BT, respectively. The XRD patterns of BT with reflections from the (200) and (002) planes are the fingerprint patterns of the tetragonal phase BT. In addition, the positions of these peaks are related to the size of the lattice. Fig. 2b shows an enlargement of the 44–46.5° range of the pattern shown in Fig. 2a. If we define the lattice constants in a conventional way, the relationship between them in the tetragonal phase is as follows:1
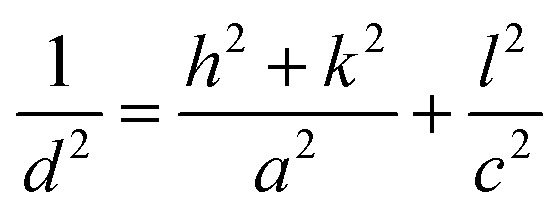


In Fig. 2b, a slightly different angle (2*θ*) of the (002) plane diffraction was observed because of the different site substitution of Er^3+^ ions into the BT host. The inter-planar spacing (*d* spacing), the lattice constants (*a* and *c*) (Å), volume of the unit cell (10^6^ pm^3^), and density (g cm^−3^) were calculated using the tetragonal phase equation (eqn (1)). The calculated parameters for A-BT and B-BT are listed in Table 2.

**Table 2:** Lattice constants (*a* and *c*), cell volume (*V*_cal_), and the calculated density of Er^3+^-doped perovskite BaTiO_3_ (*d*_cal_)

Material	Dopant ions	Ion concentration	Lattice constant and density			
			*a* (Å)	*c* (Å)	*V*_cal_ (10^6^ pm^3^)	*d*_cal_ (g cm^−3^)
t-BaTiO_3_	Er^3+^	2% (on Ba^2+^ site)	3.9799	4.0256	63.7640	6.0532
t-BaTiO_3_	Er^3+^	2% (on Ti^4+^ site)	3.9845	4.0301	63.9811	6.0738

Upon comparison, the lattice constant of B-BT is larger than that of A-BT, indicating the expansion of the unit cell in B-BT, as mentioned above. This expansion arises from the differences in the ionic sizes of Er^3+^, Ba^2+^, and Ti^4+^ (Er^3+^: 0.89 Å (with 6 coordination), Ba^2+^: 1.61 Å, Ti^4+^: 0.605 Å, O^2−^: 1.35 Å).^36–38^ In the case of doping at site A (Ba^2+^), the size of Ba^2+^ is larger than the size of Er^3+^; therefore, the lattice constant depends on the size of the O^2−^ ion rather than the size of Er^3+^. When we substitute Er^3+^ at site B (Ti^4+^), the size of Er^3+^ is larger than the size of Ti^4+^, and this causes the expansion of the unit cell, despite the low doping concentration of Er^3+^. Therefore, when comparing the diffraction patterns of A-BT with B-BT, a small peak shift is observed (44.9 *vs.* 45.0) corresponding to the (200) peak (Fig. 2b).

Fig. 2c shows the high-temperature XRD patterns of B-BT obtained at various temperatures (65–155 °C) (purple to pink, the baselines of the patterns were arbitrarily set for better visualization). We found that (a) the doublet (200)/(002) peak converged into a singlet (002) peak and (b) the doublet (100)/(001) peak merged into a singlet (001) peak. We attributed this to the phase transition from the tetragonal phase to cubic one, with an increase in temperature to ∼120 °C, the Curie temperature of the phase transition of BaTiO_3_. The transition from the tetragonal to cubic phase induces a change in the UCL intensity of Er^3+^.

**Fig. 3:**
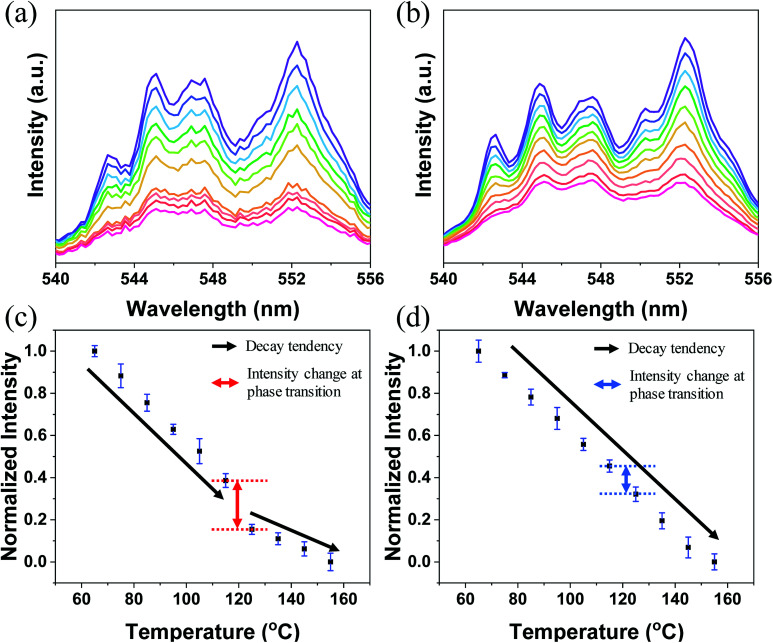
Upconversion spectra of (a) B-BT and (b) A-BT in the 540 to 556 nm range (purple to pink: 65 to 155 °C with an interval of 10 °C) and the normalized ^4^S_3/2_ → ^4^I_15/2_ emission intensity of (c) B-BT and (d) A-BT. The standard deviation error bar with the point of average normalized intensity is shown. Ten spectra were recorded at each temperature.

The log(*I*) *versus* log(*P*) plots (here, *I* denotes the UC intensity and *P* denotes the laser power) with slopes of nearly 2 (Fig. S3†) indicate that the ^2^H_11/2_ → ^4^I_15/2_, ^4^S_3/2_ → ^4^I_15/2_, and ^4^F_9/2_ → ^4^I_15/2_ transitions of Er^3+^ in both A-BT and B-BT are all two-photon upconversion processes, as depicted in Fig. S4.† In Fig. 3a and b, the upconversion emission spectra of B-BT and A-BT under 980 nm CW lasers are shown in the same temperature range (65–155 °C) as used in the high-temperature XRD experiments. Here, we focus only on the green emission (540–556 nm) because our samples show very low signal intensity in the red spectral region (∼660 nm). The temperature-dependent UC spectra of A-BT and B-BT under 980 nm CW laser irradiation were acquired at intervals of 10 °C between 65 and 155 °C. The optical setup for the temperature-dependent UCL measurement is shown in Fig. S5.† The sample powder was evenly spread on the sample holder placed on a hot plate. The focal point of the 980 nm laser was directed on the sample with the power of the laser being uniform at 340 mW. The spectra were obtained using spectrometer (HR2000+, Ocean Optics) after maintaining the temperature of the hot plate at a given temperature for 5 min to facilitate thermal equilibrium between the sample and hot plate. The reliability of the optical setup was tested by acquiring the intensity data from different positions of the samples, typically 40 times. As shown in Fig. S6b,† despite the possible heterogeneity of the powder density, standard deviation errors were not significant when the emission was measured by irradiating the laser at various locations on the sample.

Fig. 3a and c show the emission spectrum of B-BT in the 540–556 nm region and the normalized intensity of ^4^S_3/2_ → ^4^I_15/2_ emission (see Fig. S4† for the energy level diagram of Er^3+^) *versus* temperature, obtained by repeating the experiments 10 times. Fig. 3c and d are obtained by integrating the emission spectra of the region of ^4^S_3/2_ → ^4^I_15/2_ and normalizing the area to 1 for the data at 65 °C. We easily found that, in general, the intensity decreased linearly with increasing temperature. However, interestingly, we observed an abrupt attenuation of the intensity at 125 °C and a nearly linear decrease in intensity with further increase in the temperature beyond 125 °C for B-BT. As shown in Fig. S7,† the reversibility of the phase transition of B-BT was observed. The temperature, 125 °C, is higher than the Curie temperature determined by XRD analyses. In clear contrast, the UCL of A-BT (Fig. 3d) did not show any anomalous temperature dependence at high temperatures, but simply attenuated with a single slope. Therefore, we may conclude that, as B-BT undergoes phase transition from the tetragonal to cubic phase, the UCL is also affected by the phase transition. In other words, the Er^3+^ ion at the B site experiences interaction with the surrounding ions centro-symmetrically during the phase transition and deviates from the linear attenuation of UCL at lower temperatures. Subsequently, the intensity decreases linearly with a smaller slope at higher temperatures up to 155 °C.

**Fig. 4:**
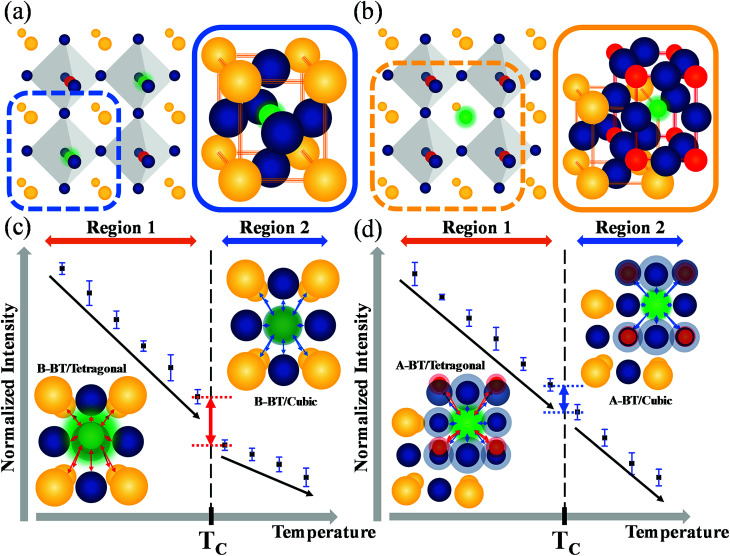
Illustrations of the unit cells of (a) B-BT and (b) A-BT along with the crystal structure (yellow: Ba^2+^, blue: O^2−^, green: Er^3+^, red: Ti^4+^) and temperature-dependent normalized upconversion intensity data with the (001) plane view of the interaction between Er^3+^ with surrounding ions in (c) B-BT and (d) A-BT. (c) Centro-symmetry distorted interaction of Er^3+^ with the surrounding ions of tetragonal B-BT at region 1 and centro-symmetric interaction of Er^3+^ with the surrounding ions of cubic B-BT at region 2. (d) Centro-symmetry distorted interaction of Er^3+^ with Ti^4+^ ions and centro-symmetric interaction of Er^3+^ with O^2−^ ions of tetragonal A-BT at region 1 and centro-symmetric interaction of Er^3+^ with the surrounding ions of cubic A-BT at region 2. Arrows represent the interaction between the ions with red for centro-symmetry distorted interaction and blue for the centro-symmetric interaction.

Fig. 4a and b illustrate the unit cells of B-BT and A-BT crystals, respectively. Fig. 4c and d represent the interaction of Er^3+^ surrounded by the ions of B-BT and A-BT, respectively. In Fig. 4c and d, regions 1 and 2 are the temperature ranges below and above the Curie temperature. Further, in Fig. 4c, the tetragonal structure of B-BT with (001) plane view is shown. Because of the structure of the tetragonal phase of BT, with a body-centered ion that deviates from the center, the interaction between the emitter Er^3+^ ion and the surrounding ions (face-centered O^2−^ and unit cell edge Ba^2+^) is not centro-symmetric. In this case, despite the parity-forbidden 4f–4f transition nature of Er^3+^, the intensity of the UCL tends to be high. In contrast, in the cubic state of B-BT at region 2, Er^3+^ occupies the center of the cubic unit cell. Thus, the interaction becomes centro-symmetric, which reduces the intensity of UCL. In other words, the system with distorted centro-symmetry, wherein the Laporte forbidden selection rule is relaxed, leads to the enhancement of the emission intensity. However, when the system transitions to the cubic phase, the luminescence intensity decreases substantially. In Fig. 4d, the (001) plane view of the tetragonal phase of A-BT is shown. In this case, the biased Ti^4+^ and Er^3+^ have symmetry-distorted interactions and the interaction of O^2−^ with Er^3+^ is centro-symmetric. The temperature-dependent UCL, which tends to decrease in region 1 (65–115 °C) with the tetragonal phase, shows the same linear attenuation for both A-BT and B-BT. However, the UCL of A-BT and B-BT are different at the Curie temperature: the slope of intensity attenuation of A-BT remains constant at regions 1 and 2. In Fig. 4d, the interactions of Er^3+^ with surrounding ions in tetragonal A-BT are shown. Compared to B-BT, A-BT has a larger number of surrounding ions (12 O^2−^ and 8 Ti^4+^). However, O^2−^ and Ti^4+^ interact with Er^3+^ in different ways: O^2−^ and Er^3+^ interact centro-symmetrically, whereas Ti^4+^ and Er^3+^ interact non-centro-symmetrically. In this way, while there are two different interaction factors in A-BT, the ionic radius of Ti^4+^ (0.605 Å) is significantly smaller than that of Ba^2+^ (1.61 Å) or O^2−^ (1.35 Å). Therefore, regarding symmetry with one ion at the center, the effects of Ba^2+^ and O^2−^ on the surrounding ions are greater than those of Ti^4+^ affecting the surrounding ions. In A-BT, the centro-symmetry distorted interaction between Er^3+^ and Ti^4+^ is insufficient to induce the crystal structure effect on the UCL, whereas, in the case of B-BT, Ba^2+^ and O^2−^ with large ionic sizes have a significant distorted centro-symmetric interaction with Er^3+^. Consequently, in tetragonal A-BT, only the Er^3+^ ion and Ti^4+^ ion with a relatively small ionic radius undergo a distorted centro-symmetric interaction through some translocation of Ti^4+^, and this does not lead to any significant change in the UCL.

## Conclusions

In summary, we investigated the upconversion luminescence of Er^3+^ ion-doped BT based on the idea that the symmetry-distorted interaction between Er^3+^ and the surrounding ions enhances the probability of a radiative transition (4f → 4f) in the Er^3+^ ions. The Er^3+^-doped BTs (A-BT, B-BT) were synthesized with solid-state reaction, where A- and B- represent the doping at “A site” and “B site” of the general perovskite structure, respectively. The UCL of Er^3+^ was measured over the temperature range of 65–155 °C to observe the phase-transition effects at ∼120 °C. UCL is generally less intense as the temperature increases because the rate of non-radiative phonon relaxation increases with increasing thermal energy. B-BT also showed such behavior up to the Curie temperature (∼120 °C) of BT; however, in the vicinity of the Curie temperature, the linearly attenuating intensity showed an abrupt change in the rate of decrease. On the contrary, the other sample, tetragonal A-BT, maintained its linear dependency on temperature. This distinct behavior indicates that the change in the upconversion intensities is due to the symmetry of the local crystal structure around emitter lanthanides, despite the fact that lanthanides are not affected significantly by the surrounding environment. This research gives significant insights into mechanisms for increasing the quantum efficiency of lanthanide-doped UC materials and how local crystal structure symmetry works at upconversion intensities of lanthanides.

## Conflicts of interest

There are no conflicts to declare.

## Supplementary Material

RA-009-C8RA09783F-s001
