# Supplementary material for: Effect of tetragonal to cubic phase transition on the upconversion luminescence properties of A/B site erbium-doped perovskite BaTiO3
Source: RSC Adv. 2019 Jan 18;9(5):2451–7. doi: 10.1039/c8ra09783f (PMC9059889; doi:10.1039/c8ra09783f)
Supplement: RA-009-C8RA09783F-s001 [file RA-009-C8RA09783F-s001.pdf]

Electronic Supplementary Material (ESI) for RSC Advances.
This journal is © The Royal Society of Chemistry 2019

Supporting Information

Effect of Tetragonal to Cubic Phase Transition on the Upconversion Luminescence Properties of A/B site Erbium-doped Perovskite $BaTiO_3$

Hyeongyu Bae and Kang Taek Lee*

Department of Chemistry, Gwangju Institute of Science and Technology,

Cheomdangwagi-ro 123, Buk-gu, Gwangju, Republic of Korea

*Corresponding author. E-mail: ktlee@gist.ac.kr, Tel: +82 62-715-3685

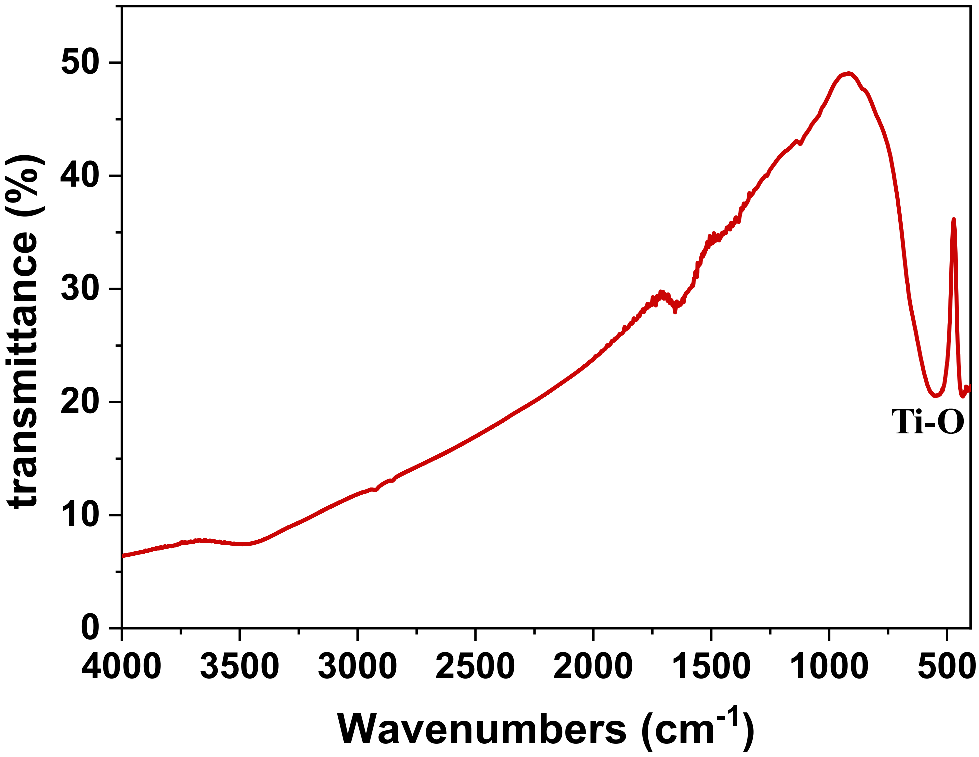

Figure S1. IR spectrum of B-BT (Ti-O vibration is the maximum phonon energy of BT).

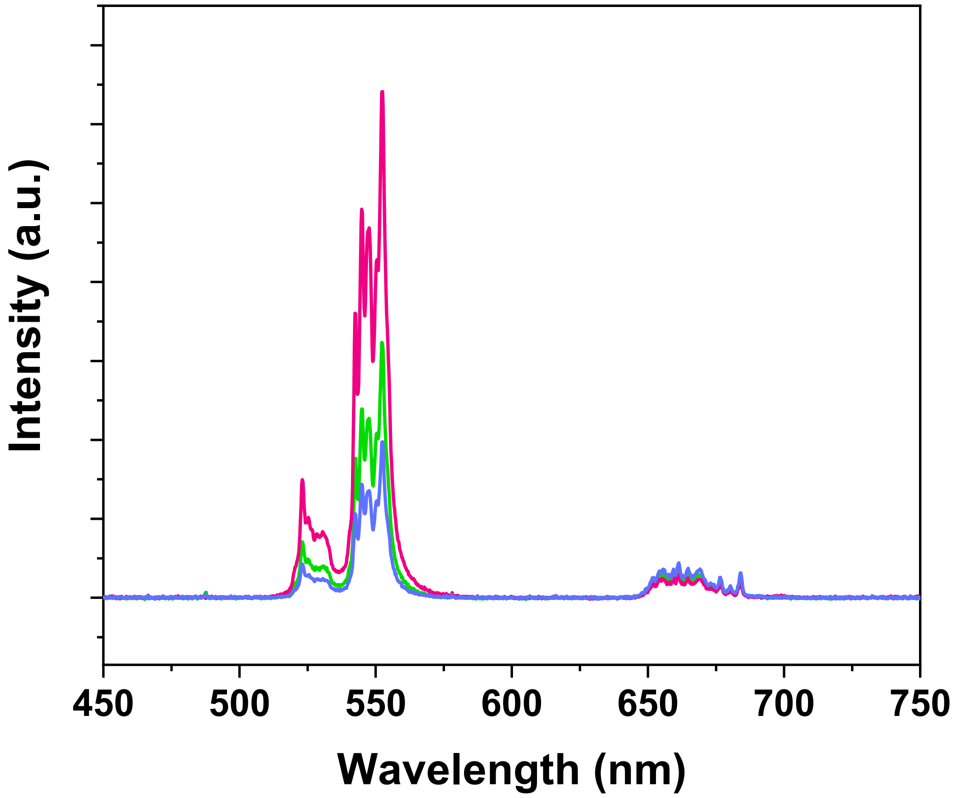

Figure S2. Upconversion emission spectrum of A-BT with different concentrations of Er^{3+} ions (green: doped with 1% Er^{3+} ion, pink: doped with 2% Er^{3+} ion, blue: doped with 3% Er^{3+} ion).

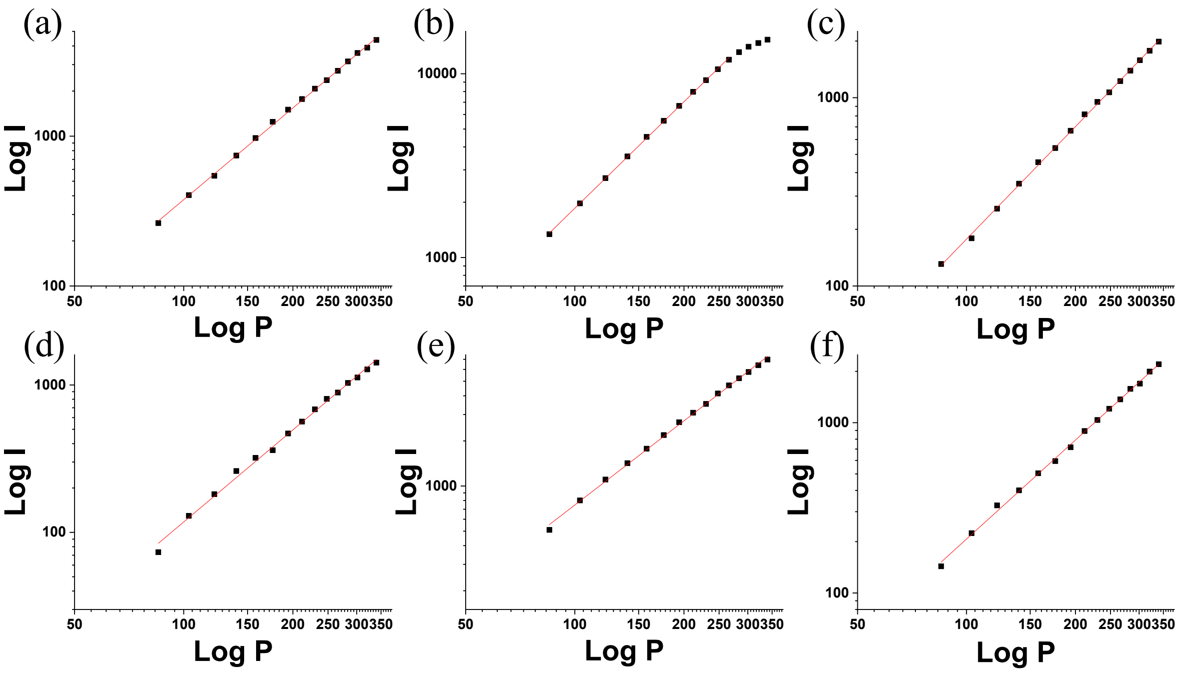

Figure S3. Log(I) versus log(P) diagram of each emission of A-BT: (a) $^2H_{11/2}\rightarrow{}^4I_{15/2}$, (b) $^4S_{3/2}\rightarrow{}^4I_{15/2}$, (c) $^4F_{9/2}\rightarrow{}^4I_{15/2}$; (a) slope = 2.03225, (b) slope = 1.92454, (c) slope = 1.98517; and log(I) versus log(P) diagram of each emission of B-BT: (d) $^2H_{11/2}\rightarrow{}^4I_{15/2}$, (e) $^4S_{3/2}\rightarrow{}^4I_{15/2}$, (f) $^4F_{9/2}\rightarrow{}^4I_{15/2}$; (d) slope = 2.07297, (e) slope = 1.86522, (f) slope = 1.9305.

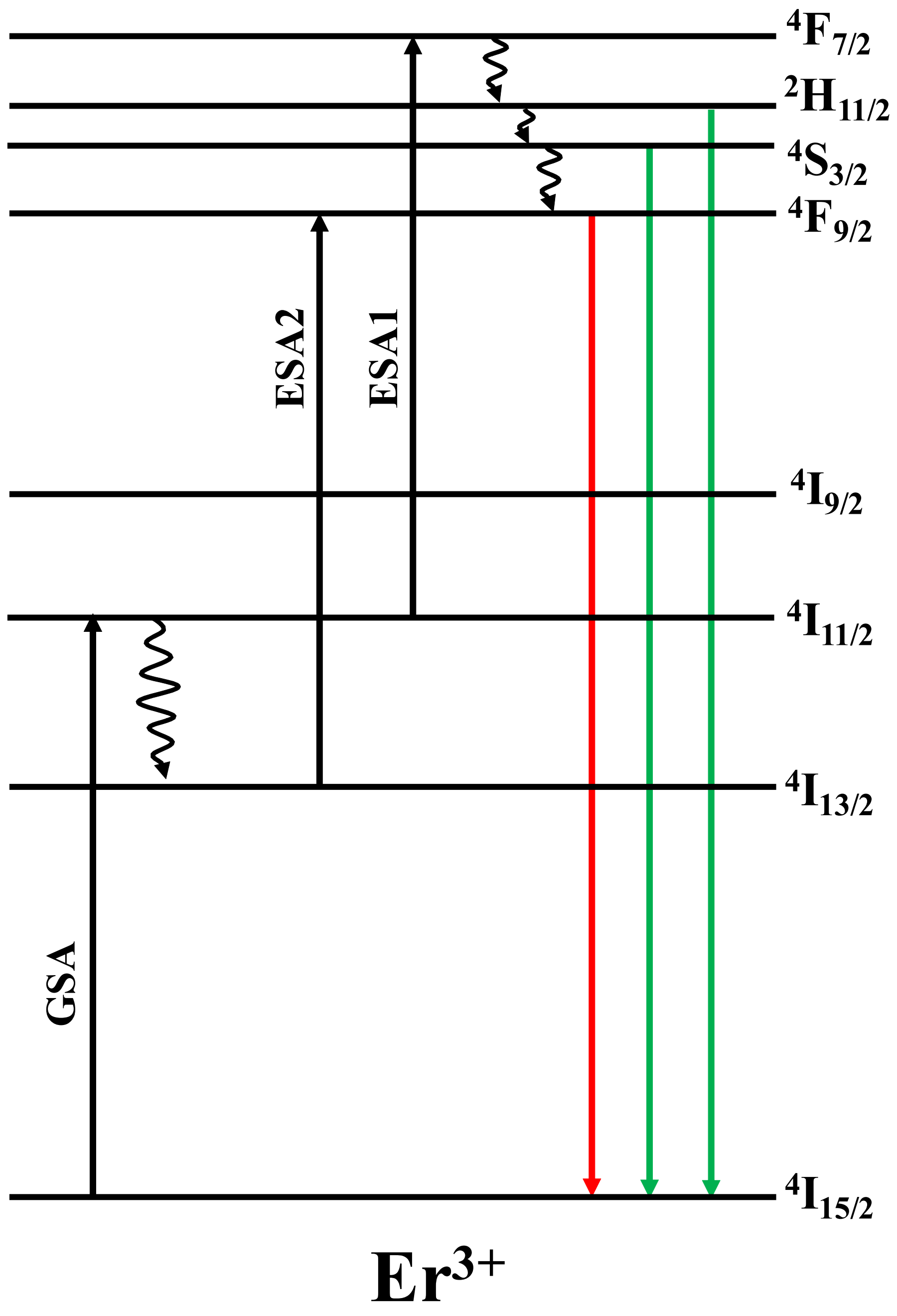

Figure S4. Schematic energy level diagram of erbium ions (Er^{3+}) (GSA: ground-state absorption, ESA: excited-state absorption).

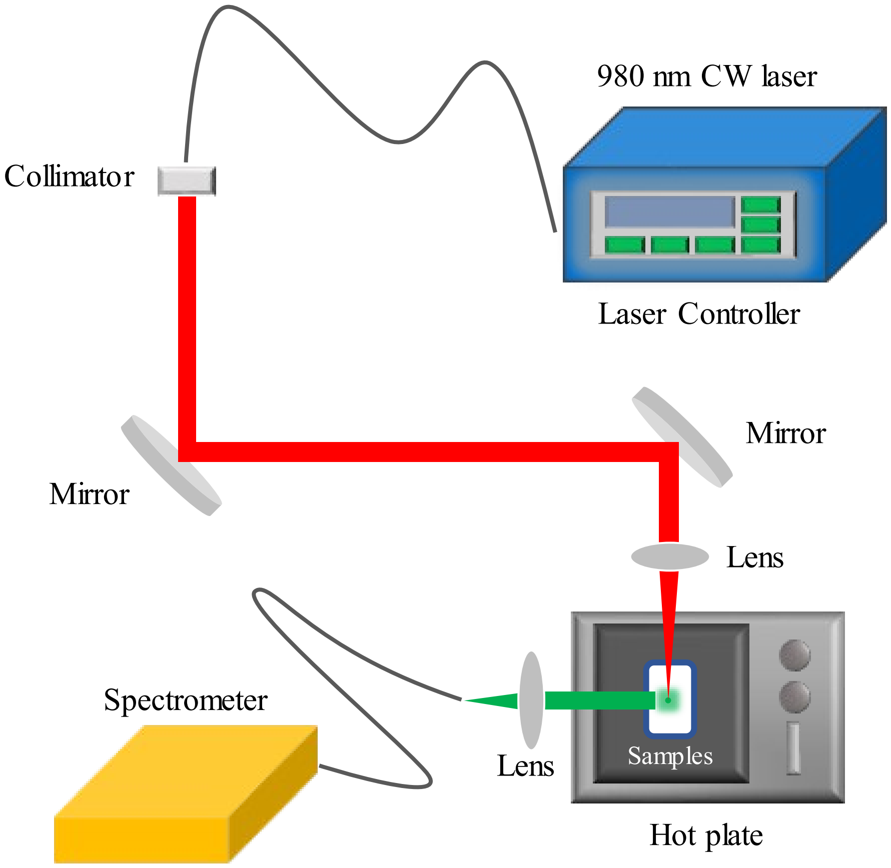

Figure S5. Optical setup for temperature-dependent upconversion spectrum measurement.

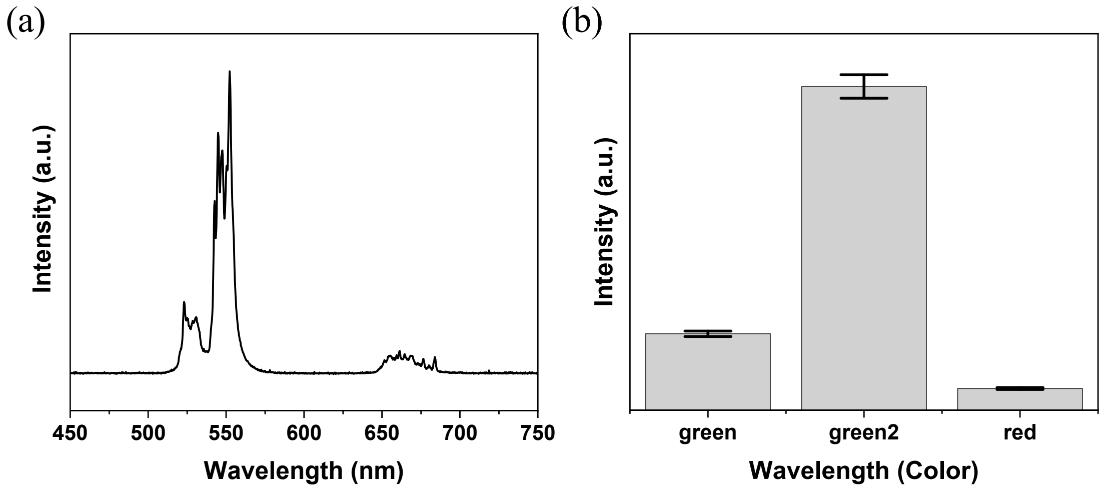

Figure S6. Optical setup reliability test. (a) Upconversion luminescence spectrum of A-BT. (b) Mean intensities of the respective luminescent points (green: $^{2}H_{11/2} \rightarrow {}^{4}I_{15/2}$, green 2: $^{4}S_{3/2} \rightarrow {}^{4}I_{15/2}$, red: $^{4}F_{9/2} \rightarrow {}^{4}I_{15/2}$), where error bars represent standard deviation (N=40).

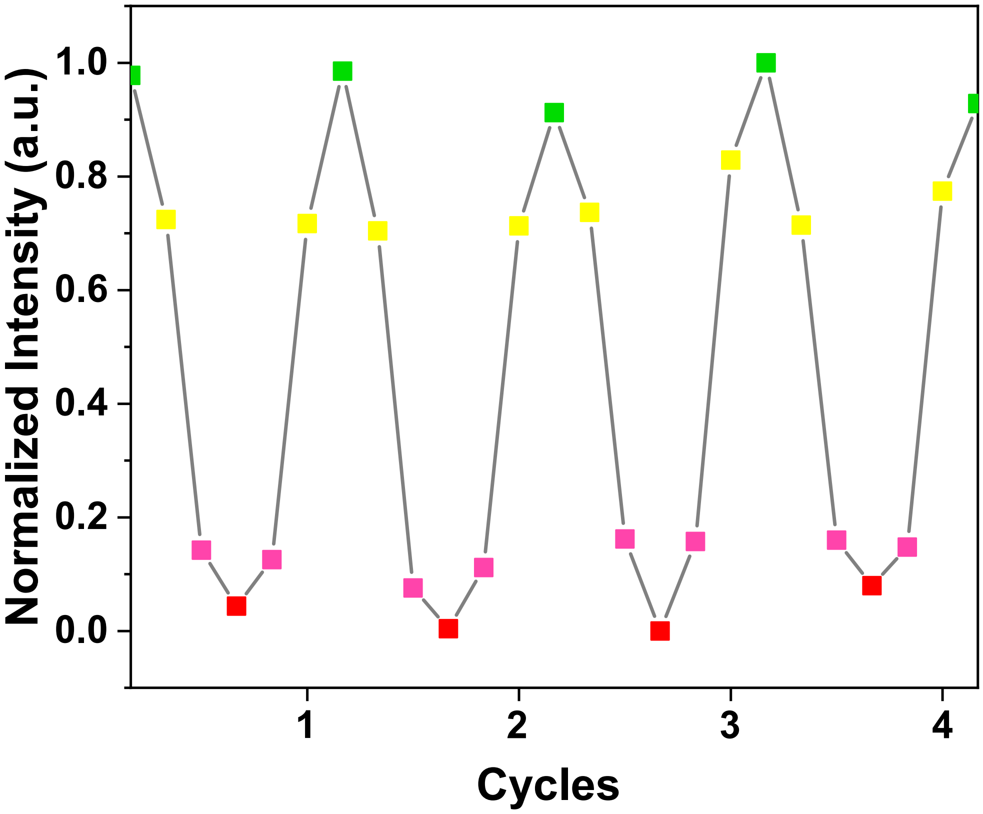

Figure S7. Normalized intensity versus cycles diagrams that show the reversibility of the B-BT intensity temperature from 105 °C to 135 °C (green: 105 °C, yellow: 115 °C, pink: 125 °C, red: 135 °C).
